# Practice Guidelines for the Value Evaluation of Clinical Pharmacy Services (version 2)

**DOI:** 10.3389/fpubh.2024.1472355

**Published:** 2025-01-14

**Authors:** Liuyun Wu, Ziyan Lv, Min Chen, Xingyue Zheng, Lian Li, Shan Du, Lizhu Han, Qinan Yin, Yin Wang, Xinxia Liu, Wenyuan Li, Xuefei Huang, Hulin Wang, Xiaoqing Yi, Xiaojiao Cui, Zhujun Chen, Yueyuan Wang, Yingying Hou, Xi Zheng, Yang Lei, Mengqiu Gou, Yue Wu, Fengjiao Kang, Fengqun Cai, Shuhong Liang, Yong Yang, Jinqi Li, Yuan Bian

**Affiliations:** ^1^Department of Pharmacy, Personalized Drug Therapy Key Laboratory of Sichuan Province, Sichuan Academy of Medical Sciences and Sichuan Provincial People's Hospital, School of Medicine, University of Electronic Science and Technology of China, Chengdu, China; ^2^Department of Pharmacy, Chengdu Women's and Children's Central Hospital, School of Medicine, University of Electronic Science and Technology of China, Chengdu, China; ^3^Department of Pharmacy, The Fourth People's Hospital of Chengdu, Chengdu, China; ^4^Department of Pharmacy, The First Affiliated Hospital of Zhengzhou University, Zhengzhou, Henan, China

**Keywords:** clinical pharmacy, pharmaceutical services, pharmaceutical practice, value evaluation, guidelines, quality management

## Abstract

**Objective:**

To optimize the construction of pharmaceutical services in medical institutions, advance the development of clinical pharmacy as a discipline, enhance the level of clinical pharmacy services, systematically implement and evaluate clinical pharmacy practices, and improve patient therapeutic outcomes, we have developed the Practice Guidelines for the Value Evaluation of Clinical Pharmacy Services (Version 2).

**Methods:**

This guideline was designed following the World Health Organization (WHO) Guideline Development Manual. The Delphi method was employed to identify clinical questions. A comprehensive systematic search was conducted to collect existing evidence on relevant issues, and the systematic reviews, evidence grading, and evidence summaries were subsequently compiled. The guideline employs the Joanna Briggs Institute (JBI) evidence level system from Australia and the Grading of Recommendations Assessment, Development and Evaluation (GRADE) system introduced by WHO in 2004 to classify the quality of evidence. Consensus on the recommendations and evidence levels was achieved through the Delphi method, resulting in the formation of the Practice Guidelines for the Value Evaluation of Clinical Pharmacy Services (Version 2).

**Results:**

Through a questionnaire survey of over 100 experts and the Delphi method voting, 23 preliminary indicators for evaluating the value of clinical pharmacy services were identified. The content of these included indicators was searched according to the PICO principle, followed by systematic reviews, meta-analyses, network meta-analyses, and related original research. Each search strategy was reviewed and approved by the guidelines steering committee. Ultimately, three dimensions for evaluating the value of clinical pharmacy were identified, encompassing 15 indicators, resulting in 20 recommendations.

**Conclusion:**

This guideline presents a set of metrics to assess the quality and effectiveness of clinical pharmacy services, which is crucial for enhancing and elevating clinical pharmacy services in healthcare institutions.

**Systematic review registration:**

http://www.guidelines-registry.org/guide/28502a74-7038-439c-bdee-d355747e2a9d, identifier: PREPARE-2022CN756.

## 1 Introduction

Since the issuance of the Opinions of the Central Committee of the Communist Party of China (CPC) and the State Council on Deepening the Reform of the Medical and Health System in April 2009 (1), China has introduced a series of policy documents aimed at transforming pharmaceutical care management. These include the Notice on Strengthening Pharmaceutical Care Management and Transforming Service Models (2), the Opinions on Accelerating the High-Quality Development of Pharmaceutical Care Services (3), and the Notice on Strengthening Pharmaceutical Care Management in Medical Institutions to Promote Rational Drug Use (4). These policies focus on transforming pharmaceutical service models, accelerating the high-quality development of pharmaceutical services, strengthening pharmaceutical discipline construction, enhancing pharmaceutical talent cultivation, and improving drug safety management. They vigorously promote innovation in pharmaceutical services and continuously enhance the level of rational drug use in clinical settings.

Over the past decade, clinical pharmacy in China has steadily progressed through continuous reforms and development, with pharmacists playing an increasingly vital role. Hospital pharmacy services are currently undergoing a transformational phase, shifting from a focus on ensuring medication supply to a patient-centered approach. This transition is marked by a shift from medication dispensing as the core function to clinical pharmacy services, and from a primary emphasis on experimental research to active involvement in clinical medication practices and promotion of rational drug use (5). With the continuous deepening of healthcare system reforms in China and the intensification of urban population aging, the demand for pharmacists in China has significantly increased, particularly in primary healthcare institutions where there is a shortage of pharmacists. Pharmacists apply their pharmaceutical expertise in conjunction with clinical knowledge to explore patterns of medication use, participate in the formulation of medication treatment plans of specified individuals, and provide medication guidance to patients. However, the value of clinical pharmacy services extends beyond these roles (6). Demonstrating the role and status of pharmacists in healthcare institutions has become a major concern for medical professionals in China.

This guideline aims to clarify the scope of clinical pharmacy services and delineate the pathways for realizing the value of these services. It seeks to transform the traditionally subjective assessments of the quality and efficacy of clinical pharmacy services in healthcare institutions into standardized processes with objective evaluation methods and criteria. By establishing scientific, standardized, and objective comprehensive evaluation procedures for clinical pharmacy services, the guideline intends to facilitate reasonable improvements and innovations in the operational models of institutional pharmacy services. This approach is expected to enhance the efficiency of pharmacists' work, fully reflect the value of their contributions, elevate the professional status of pharmacists, and promote the high-quality development of clinical pharmacy services.

## 2 Methods

### 2.1 Scope of the guideline

The guideline is applicable to healthcare institutions at all levels that provide clinical pharmacy services. The intended users of the guideline include administrative personnel of healthcare institutions, pharmacy service providers such as pharmacists and clinical pharmacists, as well as recipients of pharmacy services including clinicians, nurses, and patients.

### 2.2 Guideline development process

The Practice Guidelines for the Value Evaluation of Clinical Pharmacy Services (Version 2) was initiated by the Sichuan Academy of Medical Sciences & Sichuan Provincial People's Hospital in January 2021. It has been registered on the International Practice Guidelines Registry Platform (http://www.guidelines-registry.org/) with the registration number PREPARE-2022CN756. The development process followed the procedures outlined in the WHO's Guideline Development Manual.

The guideline team consists of the Guideline Steering Committee, Expert Group, Secretariat, and Writing Group. The Steering Committee primarily determined the theme and scope of the guideline, forms relevant groups, manages conflict of interest declarations, and approved the recommendations and the full text of the guideline. The Expert Group was responsible for defining the clinical questions of the guideline, including the population, interventions, comparisons, and outcomes (PICO), and providing input for the draft. For issues lacking definitive evidence or contentious matters, consensus recommendations were reached through the Delphi method. They proposed clinical pharmacy service value evaluation indicators to be included in the guideline based on literature review and analysis, which were ultimately confirmed through Delphi scoring. The Secretariat was responsible for systematically searching for evidence related to the questions, integrating the evidence, assessing its quality, and drafting evidence summaries. The Writing Group composed the guidelines based on the analysis reports provided by the Secretariat.

This guideline predominantly incorporates evidence from systematic reviews and randomized controlled studies (RCTs), pre-ranked according to the Joanna Briggs Institute (JBI) evidence hierarchy system for Evidence-Based Healthcare in Australia. Evidence derived from multiple high-quality RCTs or meta-analyses was designated as level A; a single clinical RCT or multiple non-RCTs as level B; a single non-RCT as level C; and observational studies or case reports as level D. For systematic reviews or RCTs, evidence grading followed the GRADE system (Grades of Recommendations Assessment, Development and Evaluation, Table 1), evaluating five downgrading factors—risk of bias (study limitations), inconsistency, indirectness, imprecision, and publication bias, and three upgrading factors—large effect size, dose-response relationship, and negative bias. The final evidence level was determined by adjusting the pre-classified rank: for example, level A evidence could be downgraded to level B with one downgrading factor, to level C with two factors, and to level D with three factors.

**Table 1:** GRADE evidence quality classification criteria.

	**Statements**
**Quality of evidence**	
High (A)	The evidence suggests that the true effect is likely to be close to the estimated effect. Further research is unlikely to alter this conclusion significantly
Moderate (B)	The evidence indicates that the true effect is probably close to the estimated effect, but there is a possibility of significant variation. Further research could influence the confidence in the estimate and potentially change it
Low (C)	The evidence provides limited confidence in the estimate of the effect, suggesting that the true effect may differ substantially. Further research is expected to have a substantial impact on the confidence in the estimate and is likely to alter it
Very low (D)	The evidence is insufficient to provide confidence in the estimate of the effect. The true effect is likely to differ substantially from the estimate
**Strength of recommendations**	
Strongly recommend (I)	In most instances, the benefits of the intervention outweigh its potential harms
Weakly recommend (II)	The pros and cons are uncertain or evidence of varying quality shows equivalent pros and cons

### 2.3 Literature screening

Using keywords clinical pharmacy, pharmacist, value, pharmaceutical services, as well as their variations, searches were conducted in Chinese databases including CNKI, VIP Chinese Scientific Journals Database, Wanfang Database, and English databases including PubMed, Embase, Cochrane Library, from January 1, 2017, to June 30, 2024. Inclusion criteria comprised: (1) Literature with its topic associated with pharmacist pharmaceutical services, drug interventions, drug counseling, drug review, medication therapy management, participation in multidisciplinary teams, or the influence of pharmacist interventions on drug economics; (2) Literature with the type of experimental articles regarding randomized controlled trials, and non-experimental articles such as systematic reviews or case reports. Exclusion criteria comprised: (1) non-Chinese or non-English literature; (2) Document types such as conference papers, theses, abstracts, reviews; (3) Literature inaccessible in full text.

Literature screening involved two stages: initial screening and full-text screening, managed using Endnote X9 software. Firstly, duplicate references were removed. Titles and abstracts were then reviewed to preliminarily identify literature that meets inclusion criteria, with full texts downloaded accordingly. Subsequently, full texts were assessed against inclusion criteria for final determination. Screening at both stages was conducted by members of the Secretariat, with cross-validation. Discrepancies were resolved through discussion, and consultation with expert panel members, if necessary, for consensus.

## 3 Results

### 3.1 Literature screening results

**Figure 1:**
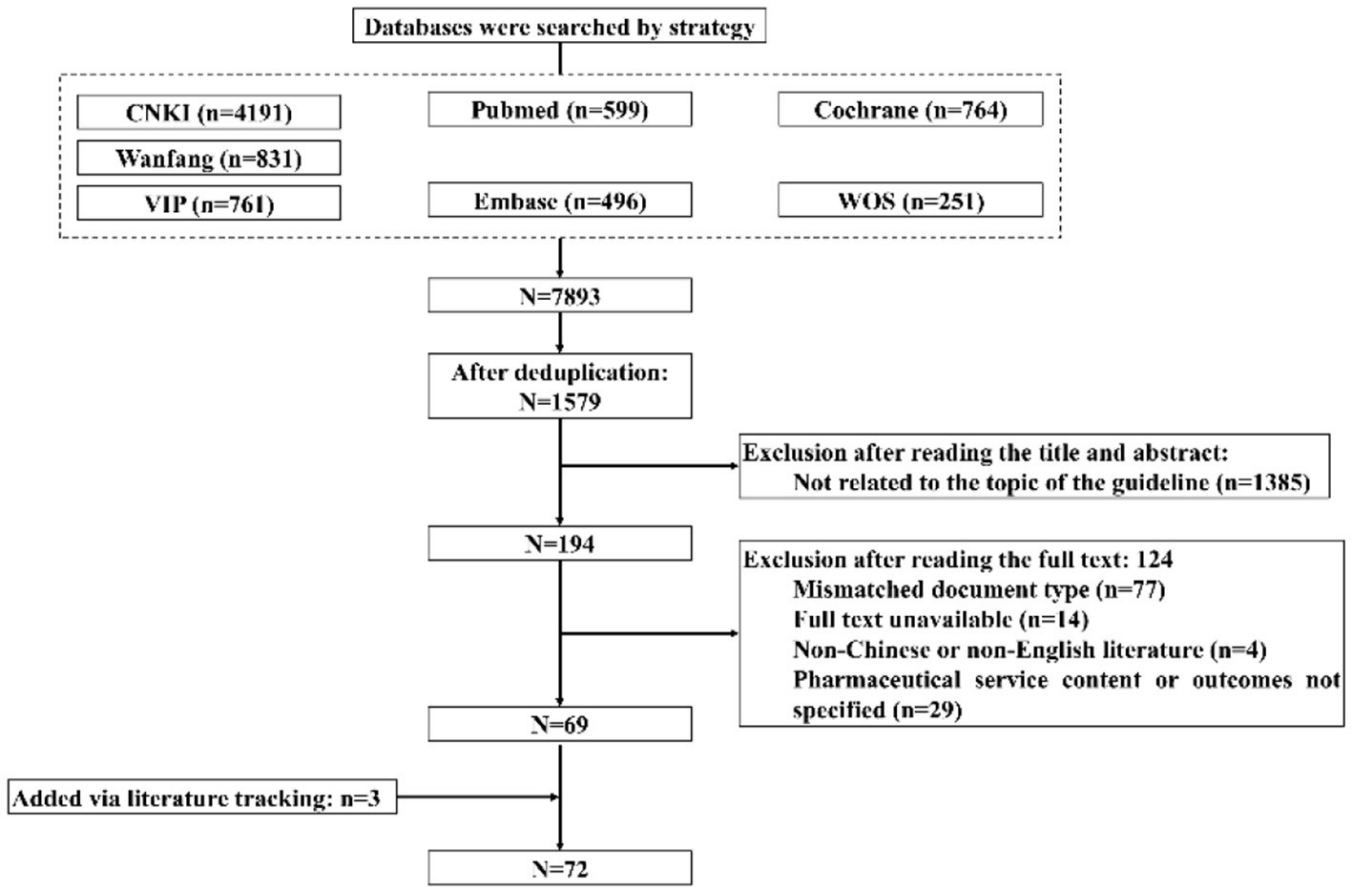
Flowchart for literature inclusion.

As of June 30, 2024, a total of 7,893 articles were retrieved. After removing duplicates, 1,579 articles remained. After screening titles and abstracts, 1,385 articles were preliminarily excluded. After full-text review, 124 articles were excluded. Additionally, three new articles were identified through literature tracking. Ultimately, 72 articles were included. The literature inclusion process is illustrated in Figure 1.

### 3.2 Identification of clinical issues

Through literature analysis, major focal points relevant to the value of clinical pharmacy services were identified, categorized, analyzed, and synthesized. Initially, 25 key clinical issues were identified. Following three rounds of Delphi survey questionnaires and two rounds of expert discussions, 20 clinical issues were finalized for inclusion in this guideline (Table 2).

**Table 2:** Summary of recommendations.

**Recommendations**	**Strength of recommendations**	**Quality of evidence**
**Clinical value**		
Recommendation 1: Pharmacists can enhance the TGAR of patients through medication review, pharmaceutical care, and evaluation of regimen appropriateness and adherence	I	A
Recommendation 2: Pharmacists can improve the TGAR of patients through medication education, lifestyle interventions	I	A
Recommendation 3: Pharmacists can reduce the patient readmission rate through medication consultations or participation in MDTs	I	A
Recommendation 4: Pharmacists can reduce patient readmission rate through care services, medication reconciliation, etc	I	A
Recommendation 5: Pharmacists can reduce ADE incidence rate through medication reviews, medication education, medication reconciliation, and participation in MDTs	I	A
Recommendation 6: Pharmacists can reduce the post-discharge MERs through medication reconciliation and/or patient education	I	A
Recommendation 7: Pharmacists can reduce the MERs in hospitalized pediatric patients through prescription review, pharmaceutical rounds, and medication education	I	B
Recommendation 8: Pharmacists can promote rational drug use in clinical practice by proactively intervening in physicians' medication decisions through prescription review and evaluation	I	B
Recommendation 9: Pharmacists can reduce MRPs and improve clinical therapeutic outcomes through MTM	I	B
Recommendation 10: Pharmacists can improve patients' medication habits and adherence through medication education	I	A
Recommendation 11: Pharmacists can expand the	I	D
scope and reach of pharmaceutical services through online remote pharmacy services, home pharmacy services, and pharmaceutical science popularization		
**Economic value**		
Recommendation 12: Pharmacists can enhance clinical outcomes and demonstrate cost-effectiveness advantages through medication rounds at admission, medication reconciliation, care during hospitalization, education and consultations at discharge, and participation in integrated clinics	I	A
Recommendation 13: Pharmacists enhance patient outcomes through medication review, education, and consultations, reducing or minimally increasing costs, demonstrating cost-utility advantages	I	A
Recommendation 14: Pharmacists reduce healthcare costs by optimizing drug therapy through clinical interventions, such as pharmaceutical care and prescription review, resulting in cost avoidance and benefit-cost advantages	I	A
Recommendation 15: Pharmacists enhance medication appropriateness in healthcare institutions through prescription review and evaluation, reducing average drug costs per outpatient and inpatient visit, thereby saving overall medication expenses and demonstrating economic value	I	B
Recommendation 16: Pharmacists reduce average hospitalization costs through individualized medication therapy and prescription order reviews	I	C
Recommendation 17: Pharmacists shorten hospital stays by participating MDTs and intervening in antimicrobial stewardship	I	A
Recommendation 18: Pharmacists enhance patient satisfaction through medication counseling, medication education, and MTM	I	B
Recommendation 19: Pharmacists enhance healthcare professionals' satisfaction through medication reconciliation, regimen optimization, pharmaceutical rounds, medication education, drug consultations, and case discussions	I	B
Recommendation 20: Pharmacists enhance patient trust through chronic disease MTM and pain management	I	B

ADE, adverse drug events; MER, medication error rate; MDT, multidisciplinary team; MRP, medication-related problem; MTM, medication therapy management; TGAR, Therapeutic Goal Attainment Rate; PAR, prescription (medical order) qualification rate.

## 4 Contents of the guideline

### 4.1 General principles

#### 4.1.1 Definition of clinical pharmacy

Clinical pharmacy integrates pharmacy with clinical practice, directly focusing on patients, centered on patient care, and is a comprehensive applied discipline that studies and practices clinical drug therapy to enhance the level of pharmacotherapy. Clinical pharmacy, with rational drug use at its core, ensures the safety, efficacy, cost-effectiveness, appropriateness, compliance, and accessibility of drug therapy.

#### 4.1.2 Contents of clinical pharmacy services

Clinical pharmacy services primarily include prescription order review and evaluation, medication reconciliation, pharmaceutical care, medication counseling, medication education and awareness, ADE monitoring, precision medicine services, medication therapy management (MTM), aiming to promote rational drug use and improve patient treatment outcomes.

#### 4.1.3 Forms of clinical pharmacy services

Forms of clinical pharmacy services include pharmacy clinics, pharmacy rounds, pharmacy consultations, MDT, home pharmacy services, internet-based remote pharmacy services, and emergency response for sudden events.

#### 4.1.4 The realization pathway of clinical pharmacy service value

**Figure 2:**
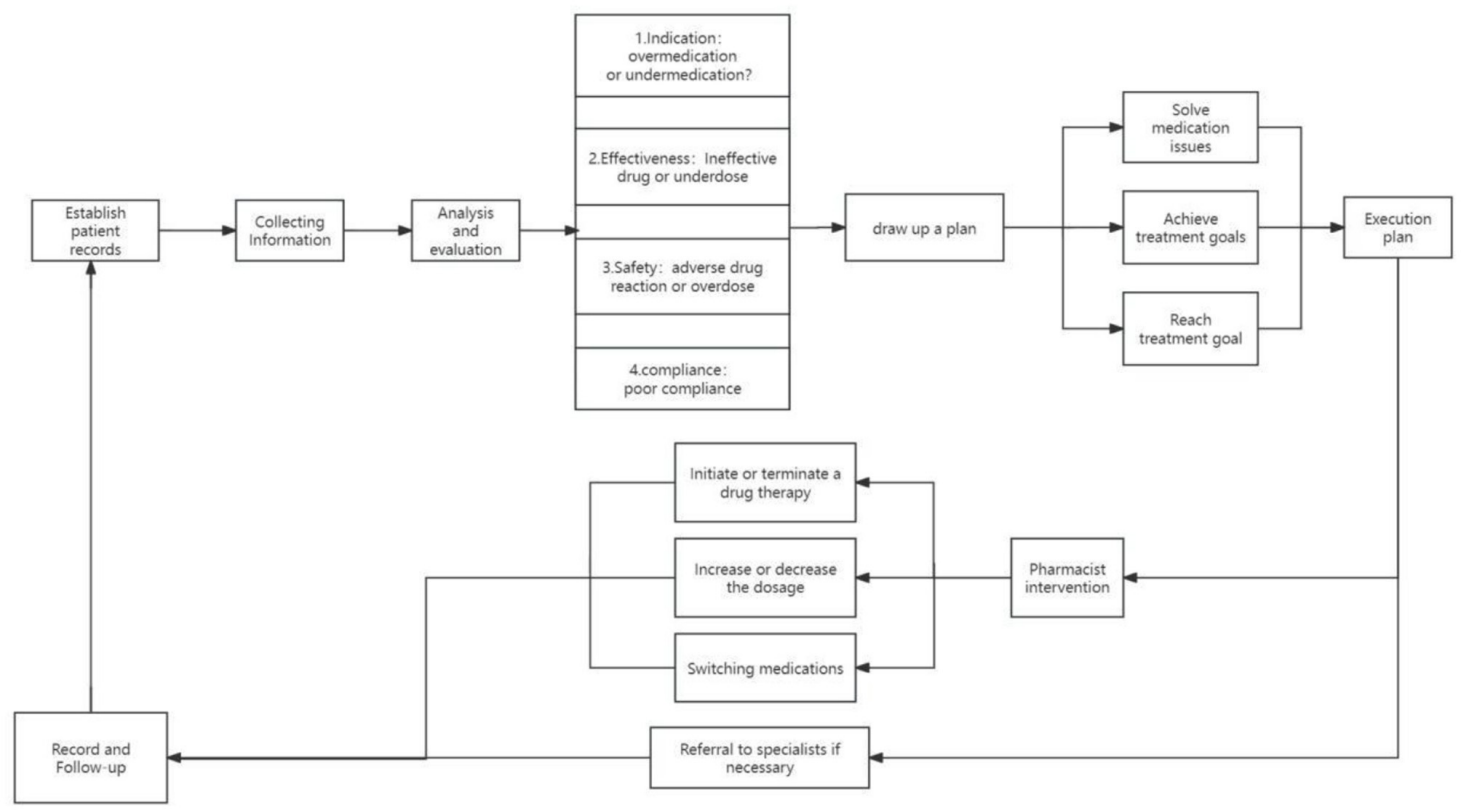
Pathways of outpatient medication therapy management.

**Figure 3:**
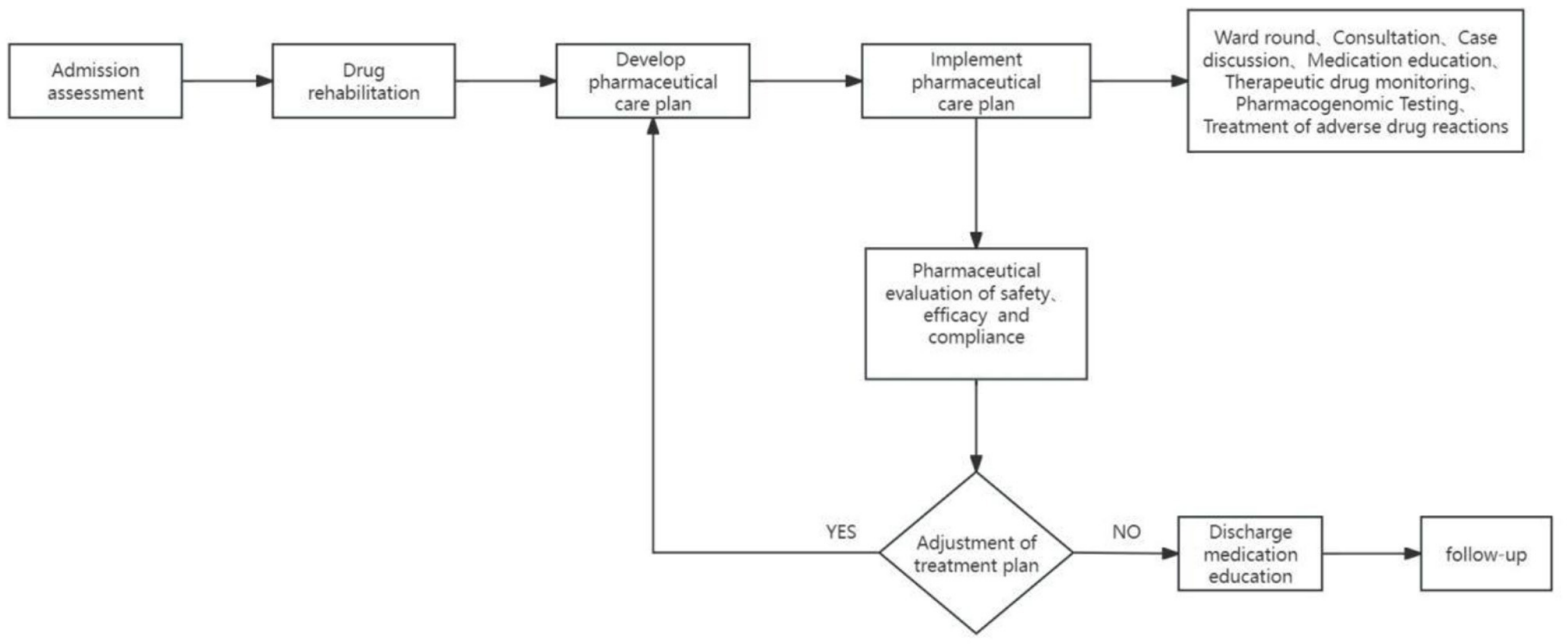
Pathways of inpatient pharmaceutical care.

**Figure 4:**
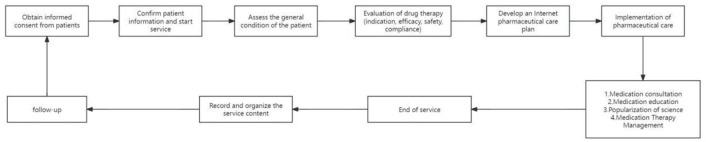
Pathway of internet remote pharmaceutical service.

**Figure 5:**
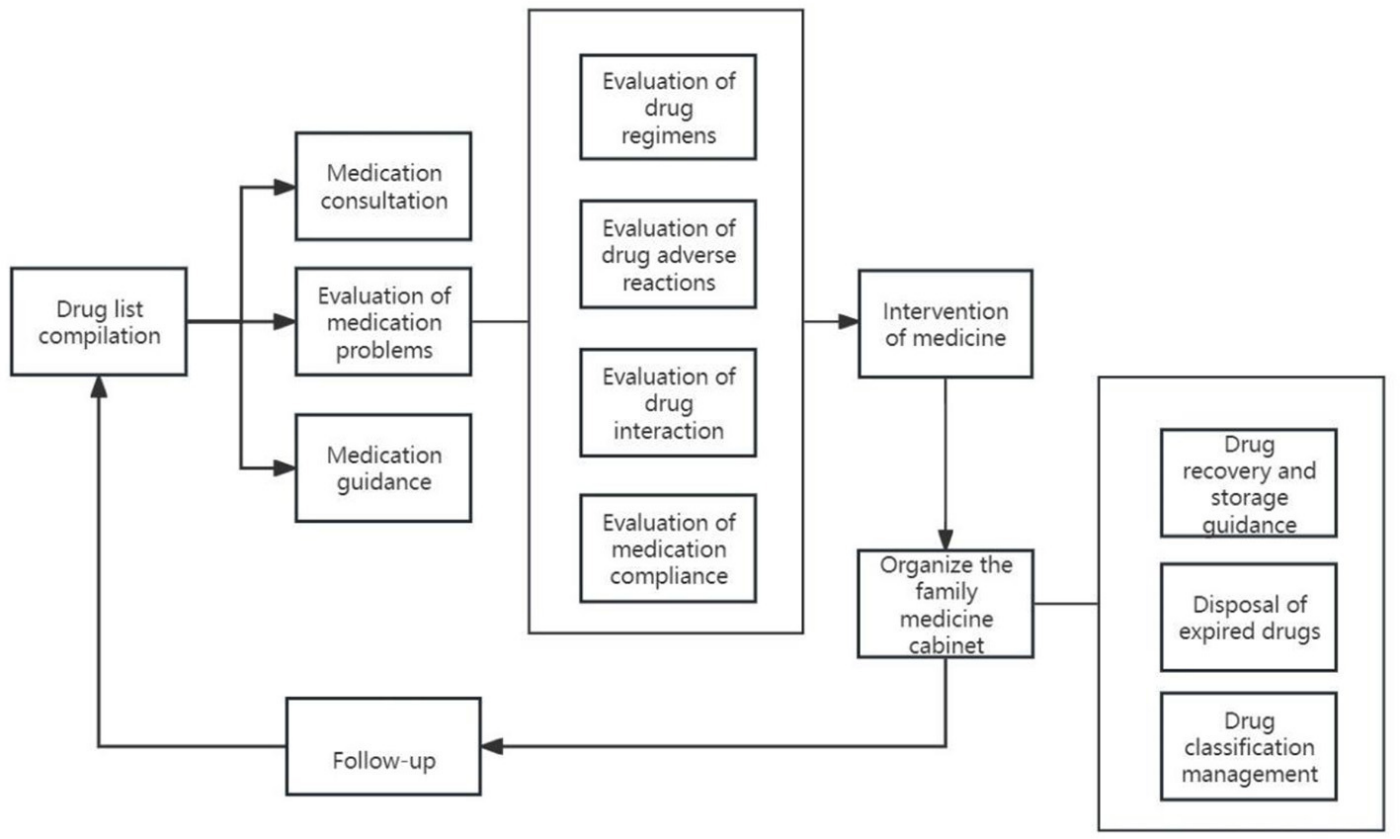
Pathway of home pharmaceutical service.

The realization pathway of clinical pharmacy service value refers to a series of steps or processes in delivering professional pharmacy services aimed at ensuring optimal drug therapy for patients, including outpatient medication therapy management pathways, inpatient pharmaceutical care pathways, internet-based remote pharmaceutical service pathways, and home pharmaceutical service pathways (Figures 2–5).

### 4.2 Evaluation of the value of clinical pharmacy services

#### 4.2.1 Clinical value assessment

(1) Therapeutic Goal Attainment Rate (TGAR)


$$\begin{array}{l} \text{TGAR}=\frac{\begin{array}{c} \text{Patients achieving treatment goals for}\\ \text{a specific disease} \end{array}}{\text{Total patients treated for a specificdisease}}\times 100\%. \end{array}$$

A meta-analysis conducted by pharmacists included 69 RCTs to investigate the impact of medication reviews on the management of cardiovascular risk factors in outpatient settings. The results showed that medication reviews significantly improved control rates for hypertension (OR = 2.73, 95% CI = 1.05–7.08), type 2 diabetes (T2DM, OR = 3.11, 95% CI = 1.17–5.88), and hypercholesterolemia (OR = 1.91, 95% CI = 1.05–3.46) (7). Another meta-analysis, which included 6 RCTs with a total of 2,573 patients, demonstrated that pharmacists' interventions, such as home blood pressure monitoring, assessment of medication regimen appropriateness and adherence, and lifestyle education, significantly improved blood pressure control in adults with chronic kidney disease (CKD) (OR = 1.53, 95% CI = 1.15–2.04, *P* < 0.01) (8).

Recommendation 1: Pharmacists can enhance the TGAR of patients through medication review, pharmaceutical care, and evaluation of regimen appropriateness and adherence (I A).

A meta-analysis of the impact of educational and behavioral interventions by medical personnel on improving the treatment outcomes of gout patients included eight studies, five of which were RCTs (2 led by pharmacists) and 3 were observational studies. Compared to the control group, patients who received medication education and behavioral interventions had a higher rate of achieving serum urate levels below 360 μmol/L (9). Another RCT also demonstrated that pharmacist involvement in providing medication education for patients with hypertension, type 2 diabetes, and hypercholesterolemia resulted in 61.7% of patients in the intervention group achieving their treatment goals, compared to only 33.3% in the control group, showing a significant contribution of pharmacist interventions to the achievement of treatment goals (10). In a retrospective cohort study, the pharmacist-physician collaborative management (PPCM) model significantly improved the target achievement rates for LDL-C (73.8% vs. 41.0%, *P* < 0.001) and heart rate (14.8% vs. 4.1%, *P* = 0.007) in post-percutaneous coronary intervention patients (11). Two studies based on diabetic populations also reported the positive impact of PPCM on achieving LDL-C targets (12, 13).

Recommendation 2: Pharmacists can improve the TGAR of patients through medication education, lifestyle interventions (I A).

(2) Readmission rate

Thirty-day readmission rate in our study refers to the ratio of the number of patients who re-admitted to the same hospital within 30 days with the same or similar primary diagnosis to the total number of discharges.

A meta-analysis encompassing 14 RCTs and 4,509 patients demonstrated that pharmacist-led medication counseling significantly reduces the 30-day readmission rate (pooled RR = 0.76, 95% CI = 0.58–0.99, *P* = 0.04) (14). Another meta-analysis led by pharmacists, which included 18 RCTs (*n* = 7,244), investigated the impact of discharge counseling on patient readmission rates and emergency department visits. The analysis revealed that pharmacist-led counseling at discharge significantly reduced patient readmission rates (RR = 0.864, 95% CI = 0.763–0.997, *P* = 0.020) (15). Several studies have examined the impact of pharmacist-provided services within MDTs on health outcomes. Among them, eight studies specifically assessed the effect of pharmacist interventions on patient readmission rates. The results suggest that the involvement of pharmacists in MDTs can reduce patient readmission rates by 32% [odds ratio (OR) = 0.74, 95% CI = 0.62–0.89] (16).

Recommendation 3: Pharmacists can reduce the patient readmission rate through medication consultations or participation in MDTs (I A).

A meta-analysis examining the impact of pharmacist interventions on readmission rates in care services included a total of 123 studies, of which 110 (89.4%) reported a reduction in readmission rates. Pharmacist interventions resulted in a decrease in readmission rates by up to 44.5%. The most common pharmacist-led intervention was medication counseling (*n* = 119, 96.7%), followed by medication reconciliation (*n* = 111, 90.2%) (17). Another meta-analysis found that care services with pharmacist interventions significantly reduced the 30-day all-cause readmission rates for patients with congestive heart failure compared to standard care (18).

Recommendation 4: Pharmacists can reduce patient readmission rate through care services, medication reconciliation, etc (I A).

(3) ADE incidence rate


$$\begin{array}{l} \text{ADE incidence rate}=\frac{\text{number of ADE cases}}{\text{total cases for evaluating ADE}}\;\times 100\%, \end{array}$$

A systematic review encompassing 23 RCTs and interventional studies indicated that pharmacist-led interventions significantly reduce ADEs in older adults in nursing homes, with the most common interventions being medication reviews and medication education (19). A meta-analysis of 14 RCTs and interventional studies demonstrated a significant association between the reduction of ADE incidence and the involvement of pharmacists in MDT care. Compared to clinical outcomes of MDTs without pharmacist involvement, MDTs with pharmacist participation had odds ratios for preventable and non-preventable ADEs of 0.26 (95% CI = 0.15–0.44; *P* < 0.00001) and 0.47 (95% CI = 0.28–0.77; *P* = 0.003), respectively (20). A 2021 Cochrane systematic review showed that pharmacist involvement in medication reconciliation reduced ADEs compared to patients without medication reconciliation (OR 0.38, 95% CI = 0.18–0.80, *n* = 1,336) (21).

Recommendation 5: Pharmacists can reduce ADE incidence rate through medication reviews, medication education, medication reconciliation, and participation in MDTs (I A).

(4) Medication Error Rate (MER)


$$\begin{array}{l} \text{MER}=\frac{\begin{array}{c} \text{Number of individuals involved in}\\ \text{medication errors} \end{array}}{\begin{array}{c} \text{Total number of patients on medication}\\ \text{during the same period} \end{array}}\times \;100\% \end{array}$$

A meta-analysis incorporating 10 RCTs indicated that pharmacist interventions, including medication reconciliation and education, significantly reduced post-discharge MERs compared to the control group (OR 0.44; 95% CI = 0.31–0.63) (22). Another meta-analysis of 6 quasi-experimental studies (*n* = 29,291) demonstrated that pharmacist interventions, such as prescriptions review, ward rounds participation, and educational sessions providing in clinical practice, effectively decreased MERs in hospitalized pediatric patients (OR 0.27; 95% CI = 0.15–0.49) (23).

Recommendation 6: Pharmacists can reduce the post-discharge MERs through medication reconciliation and/or patient education (I A).

Recommendation 7: Pharmacists can reduce the MERs in hospitalized pediatric patients through prescription review, pharmaceutical rounds, and medication education (I B).

(5) Prescription (or Medical Order) Qualification Rate (PAR)


$$\begin{array}{l} \text{PAR}=\frac{\begin{array}{c} \text{number of qualified prescriptions}\\ \left(\text{medical orders}\right) \end{array}}{\begin{array}{c} \text{total number of prescriptions }\left(\text{medical orders}\right)\\ \text{reviewed during the same period} \end{array}}\times \;100\% \end{array}$$

The PAR refers to the proportion of prescriptions (medical orders) issued by doctors that meet the criteria of safety, effectiveness, cost-efficiency, and appropriateness of medication. This rate not only reflects the diagnostic and treatment level and quality of healthcare institutions but is also an important indicator for evaluating patient medication safety. Pharmacists significantly positively impact the outcomes of patients through prescription order review and evaluation, thereby improving the rationality of clinical medication use and treatment levels (24). In a retrospective study, pharmacists intervened in 38.2% of the prescriptions (medical orders) reviewed, resulting in a significant positive impact on patients and preventing medication errors to some extent (25). In another retrospective study involving 717 patients, pharmacist intervention significantly reduced the usage rate of proton pump inhibitors (PPIs), decreasing the incidence of irrational drug use by 45.8%, and significantly improving the rationality of PPI use (26). Pharmacists can enhance the rationality of clinical medication use and treatment levels through proactive intervention in medication practices.

Recommendation 8: Pharmacists can promote rational drug use in clinical practice by proactively intervening in physicians' medication decisions through prescription review and evaluation (I B).

(6) The Number of medication-related problems

Medication-related problems (MRPs) encompass seven major categories: unnecessary drug therapy, the need for additional drug therapy, ineffective drugs, subtherapeutic dosage, adverse drug reactions (ADRs), excessive dosage, and patient non-compliance. In 2003, the United States Medicare Prescription Drug, Improvement, and Modernization Act incorporated medication therapy management (MTM) into the Medicare prescription drug benefit program, leading to its widespread implementation (27). In a pharmacist-led MTM service provided to nursing home residents, pharmacists identified a total of 675 MRPs, with the most common issues of unnecessary drug therapy (22%), excessive dosage (17%), and ADRs (16%). Physicians accepted 64% of the pharmacists' recommendations, contributing to the immediate resolution of 32% of the MRPs, thereby significantly enhancing clinical outcomes (28). In another study, pharmacists participated in the MDT for cancer pain management and identified 12 MRPs. The top three issues were poor adherence (27.69%), inappropriate drug selection (22.56%), and improper dosing (16.41%). Following pharmacist interventions, these MRPs were reduced by 74.54%, and patients' pain scores significantly decreased from 2.80 ± 1.92 to 1.90 ± 1.58 (*P* < 0.05), demonstrating the positive impact of the MTM services of pharmacists (29).

Recommendation 9: Pharmacists can reduce MRPs and improve clinical therapeutic outcomes through MTM (I B).

(7) Adherence

Medication adherence refers to the extent to which patients follow their prescribed medication regimens. Adherence scales are the primary quantitative indicators used to assess patients' medication adherence and are crucial for evaluating the effectiveness of drug therapy. The most widely used scale is the Morisky Medication Adherence Scale (MMAS) (30, 31), along with some specialized modified scales, such as the Hill-Bone Compliance Scale for antihypertensive medication and the Medication Adherence Report Scale (MARS) for psychiatric patients (32, 33). A meta-analysis incorporating 12 RCTs compared the impact of pharmacist interventions on medication adherence in patients with chronic obstructive pulmonary disease (COPD). Among these studies, two used the medication adherence rating scale, four used MMAR-4, and one used MMAR-8 to assess adherence. The results indicated that pharmacist-led medication interventions could improve inhalation techniques in adult patients with asthma or COPD, with a significant improvement in medication adherence particularly observed in COPD patients (34). Another meta-analysis, which included 59 RCTs, aimed to evaluate the impact of pharmacist-led interventions on the prognosis of patients with diabetes. The findings revealed that patient education, training, or discussions led by pharmacists more effectively improved medication adherence compared to other interventions. Following these interventions, patients exhibited significantly lower blood glucose levels and enhanced therapeutic outcomes (35).

Recommendation 10: Pharmacists can improve patients' medication habits and adherence through medication education (I A).

(8) The application scenarios and impact scope of pharmaceutical services

Pharmacists continuously expand the scope of pharmaceutical services through various means, extending the reach of these services (36). During major infectious disease outbreaks and public health emergencies, internet-based remote pharmaceutical services can effectively ensure that the public has timely access to pharmaceutical care, thereby improving the accessibility of healthcare services (37). Pharmacists can provide pharmaceutical services via internet-based telemedicine and manage transitional care for discharged patients by conducting comprehensive medication reviews, determining medication lists, identifying MRPs, and offering intervention suggestions to patients. This expansion broadens the scenarios and reach of pharmaceutical services (38). The deep integration of information technology and artificial intelligence with clinical pharmaceutical services is continually expanding the application scenarios and reach of pharmaceutical care.

Recommendation 11: Pharmacists can expand the scope and reach of pharmaceutical services through online remote pharmacy services, home pharmacy services, and pharmaceutical science popularization (I D).

#### 4.2.2 Economic value assessment

From a healthcare system perspective, costs encompass direct medical expenses such as medication treatments and physical therapies related costs, pharmaceutical service costs, and opportunity costs associated with the resources consumed or sacrifices made to implement preventive, diagnostic, or therapeutic interventions. Pharmaceutical services may incur additional costs due to the time and human resources invested by pharmacists. However, these interventions might also reduce medication regimen expenses, potentially leading to an overall increase or decrease in total costs.

(1) Cost-Effectiveness Ratio (CER) (39).


$$\begin{array}{l} \text{CER}=\frac{\text{cost}}{\text{effectiveness}}\\ \text{ICER}=\frac{\text{Cost A}-\text{Cost B}}{\text{effectiveness A}-\text{effectiveness B}}\; \end{array}$$

The Guidelines for Pharmacoeconomic Evaluations in China (2020) state that effectiveness refers to the improvement in clinical indicators of patients resulting from pharmaceutical services. The Incremental Cost-Effectiveness Ratio (ICER) can measure whether the additional costs incurred by providing pharmaceutical services are worthwhile for the additional outcomes achieved in patients. If the ICER is below the maximum willingness-to-pay threshold of decision-makers, also the cost-effectiveness threshold, the intervention is considered cost-effective. However, due to the complexity and diversity of clinical indicators, it is challenging to determine a threshold for each specific clinical indicator. A RCT conducted in the United States investigated the joint management of patients by pharmacists and physicians. In the pharmacist intervention group, the cost to reduce systolic blood pressure by 1 mmHg was $33.27, and the cost to reduce diastolic blood pressure by 1 mmHg was $69.98. The cost to increase the hypertension control rate by one percentage was $22.55. The study results emphasize the cost-effectiveness of pharmacists in managing hypertension within primary care settings (40). A cost-effectiveness analysis of integrating pharmacists into primary care teams to reduce cardiovascular risk in T2DM patients revealed that pharmacists provided an additional (3.0 ± 1.9) hours of service per patient, encompassing medication reconciliation, medication counseling, and therapeutic medication management. The total treatment cost per patient was (226 ± 1,143) USD. Compared to usual care, the total annual healthcare utilization cost per patient in the intervention group decreased by 190 USD (95% CI = 668–1040 USD), while the annualized risk of cardiovascular events among patients decreased by 0.3% (95% CI = 0.08%−0.6%). When the annual cardiovascular risk reduction reached 1%, society was willing to pay 4,000 USD, making the intervention 95% likely to be cost-effective compared to usual care. Thus, integrating pharmacists into primary care teams is a cost-effective strategy to reduce cardiovascular risk in T2DM patients (41). A domestic cost-effectiveness study on pharmacist involvement in the treatment of community-acquired pneumonia (CAP) revealed that pharmacists' interventions, including medication education, prescription review, and pharmaceutical care, significantly reduced the total treatment cost in the experimental group (9,975.09 CNY vs. 15,656.86 CNY) and improved clinical efficacy (96.67% vs. 85%), demonstrating favorable cost-effectiveness (CER 10,318.7 vs. 118,419.84) (42). Erinn et al. conducted a pharmacist-led medication appropriateness review for three different severity levels of ADEs and performed a cost-effectiveness analysis of clinical pharmacy services. The results indicated that the medication appropriateness review saved €27,651.82 for 337 high-risk inpatients with opioid-induced persistent constipation, €4,995.23 for 131 high-risk inpatients with ketorolac-associated upper gastrointestinal bleeding, and €4,655.723 for 420 high-risk inpatients with drug-induced torsades de pointes ventricular tachycardia. The ICER were 301.11, 3,669.46, and 8,846.13, respectively (43).

Recommendation 12: Pharmacists can enhance clinical outcomes and demonstrate cost-effectiveness advantages through medication rounds at admission, medication reconciliation, care during hospitalization, education and consultations at discharge, and participation in integrated clinics (I A).

(2) Cost-Utility Ratio (CUR)


$$\begin{array}{l} \text{CUR}=\frac{\text{cost}}{\text{utility}}\; \end{array}$$

The ultimate goal of pharmaceutical services is to enhance patient health benefits, specifically health utility (U). In cost-utility analysis, health outcomes are measured in terms of Quality Adjusted Life Years (QALYs), which represent the number of years lived in full health, adjusted according to the number of years of life gained by a patient in a particular living state with the measurement of a specific weight. According to the China Guidelines for Pharmacoeconomic Evaluations (2020), cost-utility analysis also reports the ICER value, which indicates the incremental cost required to gain one additional QALY when comparing the target intervention to a control intervention (39).

A systematic review evaluated the economic impact of pharmacist-led medication reviews in outpatient settings for patients with hypertension, T2DM, and dyslipidemia (44). The review included a total of 11 RCTs, of which eight studies concluded that medication reviews were cost-effective. Additionally, two studies conducted cost-utility analyses, reporting ICERs of $612.7 and $59.8 per QALY, respectively. These findings indicate that medication reviews offer economic advantages in the pharmacological treatment of various diseases. A RCT conducted among older adults confirmed that pharmacist-led patient education and counseling services can reduce the inappropriate long-term use of medications (45). Compared to routine care, the enhanced pharmaceutical services saved $1,008.61 in costs while increasing QALYs by 0.11, demonstrating economic efficiency. Another study evaluated the long-term costs and clinical outcomes of a collaborative intervention by pharmacists and physicians vs. physician-only management in the treatment of primary hypertension (46). The study found that the collaborative intervention increased QALYs by 0.14 with an ICER of $26,807.83 per QALY, which is below the payment threshold of per capita GDP. High-risk patients benefited the most from the collaborative intervention, suggesting that the pharmacist-physician collaboration is a cost-effective strategy in hypertension treatment. Similar study has analyzed the cost-effectiveness of pharmacist-led medication therapy management vs. routine management in reducing cardiovascular risks in hypertensive patients (47). Compared to the routine management group, patients in the intervention group saved an incremental cost of 4,770 (Canadian dollar) and gained an additional 0.18 QALYs, indicating that pharmacist-led interventions not only reduce treatment costs but also yield greater benefits. Another study analyzed the cost-effectiveness of pharmacist involvement in outpatient chemotherapy for breast cancer (48). Pharmacists provided medication consultations and education services, monitored ADRs of chemotherapy drugs, and offered coping suggestions. The hourly wage for pharmacists providing these services was JPY 2,227, with an additional annual cost of JPY 37,859 and a maximum QALY change of 0.007 ± 0.199. The maximum calculated ICER for pharmaceutical consultations was USD 12,460 per QALY, which is below the per capita GDP payment threshold. This pharmaceutical consultation service helps improve patients' quality of life without significantly increasing healthcare costs, demonstrating economic viability. Cost-effectiveness analyses have proven that pharmaceutical consultation services have an economic advantage and are worth promoting widely to benefit more patients.

Recommendation 13: Pharmacists enhance patient outcomes through medication review, education, and consultations, reducing or minimally increasing costs, demonstrating cost-utility advantages (I A).

(3) Benefit-Cost Ratio (BCR)


$$\begin{array}{l} \text{BCR}=\frac{\text{the overall benefits of pharmacist services}}{\text{the total costs of pharmacotherapy and related services}} \end{array}$$

In the management of adult patients in the ICU, pharmacist interventions, including ADR prevention (18%), individualized pharmaceutical monitoring (36%), bedside monitoring (23%), and management and supportive tasks (drug evaluations) (13%), result in an annual total cost savings of $263,500, with a BCR of 24.2:1 (49). In the management of treatment-related issues for chronic disease outpatients, the cost of pharmacists conducting home medication management is $1,078 per month, with a total benefit of $6,444 per month, yielding a BCR of 5.98:1 and an annual net benefit of $64,393 (50). In the management of pediatric patients, pharmacist-led pharmaceutical services have been shown to reduce hospitalization costs by 30.0% and medication costs by 33.7%. The cost of clinical pharmacy services is CNY 184.1 per person, with BCR of 9.45:1 and 4.61:1, respectively, demonstrating economic viability (51). A study evaluating pharmacist-physician collaboration for medication management counseling in discharged patients found that at 12 months, the readmission rate for the control group was 1.65 per person-year, while the readmission rate for the intervention group was 1.09 per person-year [incidence rate ratio (IRR) = 0.79; 95% CI = 0.52–1.18]. Additionally, the emergency visit incidence rate (IRR = 0.46; 95% CI = 0.22–0.94) and the combined readmission and emergency visit incidence rate (IRR = 0.69; 95% CI = 0.48–0.99) were significantly reduced. The incremental net cost-benefit of pharmacist intervention was $5,072 per patient, with a BCR of 31:1 (52). Additionally, the use of mobile programs for health management by pharmacists for therapeutic drug management in kidney transplant patients significantly reduced hospitalization rates (1.08 vs. 0.65 per person-year, *P* = 0.007) and hospitalization costs (RR: 0.51, 95% CI = 0.28–0.91; *P* = 0.022), with an estimated net cost saving of $368,839 and a return on investment (ROI) of $4.30 for every dollar spent (53). Beyond specific specialties, the implementation of pharmacists' review over clinical medication orders hospital-wide demonstrates a positive BCR. Over five consecutive working days, 14 hospital pharmacists and three hospital pharmacy residents conducted 622 medication order reviews for 558 patients, identifying 709 MRPs. The primary recommendation by clinical pharmacists was to discontinue medication (38.6%), preventing 59.07 adverse drug events and saving a total of €8,659.54 per week (54).

A systematic review based on cost-benefit analysis of the economic value of pharmaceutical services in medical institutions in China included 46 studies from 17 countries, predominantly from the United States (21.74%), China (19.57%), and France (8.70%). Among these, 25 studies were prospective in design. The types of pharmaceutical services covered in the included literature were primarily pharmaceutical care (60.87%), followed by prescription review (23.91%), medication reconciliation (8.70%), and pharmacy clinics (6.52%). The analysis results showed a median BCR of 5.05 (3.08–11.28) for pharmaceutical services. Studies from Belgium and Chile reported the highest BCR for pharmaceutical services, at 25 and 24.2, respectively; followed by Ireland at 16.54 (12.59–18.63); and China ranked fourth with a BCR of 9.45 (6.83–13.70). Studies from Australia and Thailand yielded lower BCRs of 2.19 and 2.83, respectively. Subgroup analysis by type of pharmaceutical service revealed BCRs of 6.55 (5.13–13.64) for pharmacy clinics, 5.98 (4.20–12.65) for prescription review, 5.50 (2.11–11.02) for medication reconciliation, and 4.70 (3.03–10.4) for pharmaceutical care, with no statistically significant differences (*P* = 0.732) (55).

Recommendation 14: Pharmacists reduce healthcare costs by optimizing drug therapy through clinical interventions, such as pharmaceutical care and prescription review, resulting in cost avoidance and benefit-cost advantages (I A).

(4) Total effect

1) Average drug costs per visit


$$\begin{array}{l} \text{Average Drug Cost per Outpatient Visit}\\ \;=\frac{\text{Outpatient Drug Costs}}{\text{Number of Outpatient Visits}}\\ \text{Average Drug Cost per Discharged Patient}\\ \;=\frac{\text{Discharged patient Drug Costs}}{\text{Number of Discharges}} \end{array}$$

The Operational Manual for Performance Assessment of Tertiary Public Hospitals (2023 Edition) (56) indicates the implementation of a Chief Pharmacist system in tertiary general hospitals (57). The primary interventions under this system include reviewing the rationality of medication use across the entire hospital and providing patient-centered pharmaceutical services. These measures have resulted in an average reduction of $34.3 million in total drug costs, $8.9 in outpatient drug costs per visit, and $303.9 in inpatient drug costs per admission. Furthermore, drug use indicators have significantly improved without compromising the quality of clinical treatment. A comprehensive second-class hospital has improved the rationality of medication use within the institution through continuous pharmaceutical interventions such as pharmaceutical management, prescription review, and medication order evaluation. Over 3 years, these interventions have led to a reduction of CNY 581 per inpatient visit on average, a decrease of 30.8% (58). Pharmacists participated in pharmaceutical rounds and medication therapy management for inpatients with acute exacerbation of chronic obstructive pulmonary disease (AECOPD). Through medication order review and recommendations, they enhanced the rational use of medications, while also providing pharmaceutical services that included basic disease knowledge, guidance on the use of inhalation preparations, and management of ADRs (59). Compared to the conventional treatment, the pharmacist intervention had significantly lower average medication costs per hospital stay (5,717 ± 449 vs. 8,002 ± 755, *P* = 0.004) and lower average antibiotic costs per hospital stay (3,639 ± 379 vs. 5,636 ± 641, *P* = 0.007). The pharmaceutical interventions significantly reduced medication expenses for AECOPD patients.

Recommendation 15: Pharmacists enhance medication appropriateness in healthcare institutions through prescription review and evaluation, reducing average drug costs per outpatient and inpatient visit, thereby saving overall medication expenses and demonstrating economic value (I B).

2) Medical costs


$$\begin{array}{l} \text{Average Cost per Discharged Patient}\\ \;=\frac{\text{Total Inpatient Costs}}{\text{Number of Discharged Patients}}\;\\ \text{Average Cost per Outpatient Visit}\\ \;=\frac{\text{Total Outpatient Costs}}{\text{Number of Outpatient Visits}}\; \end{array}$$

Multiple meta-analyses have concluded that pharmaceutical services can reduce the average medical costs for hospitalized patients. One RCT and two observational cohort studies analyzed the costs of anticoagulation management in different currency and time units, concluding that pharmacist-led individualized drug therapy, primarily focused on anticoagulation management, can reduce hospitalization costs. In this study, Chan et al. noted that after pharmacist intervention, the monthly cost per patient significantly decreased from (98 ± 158) USD to (76 ± 95) USD (*P* < 0.01) (60). Hall et al. (61) reported that, taking into account the operational costs of anticoagulation services and available medication expenses, the overall net medical cost savings per patient in the pharmacist-managed anticoagulation service group was 3,697 USD. According to the study by Hou et al. (62), the total cost (including anticoagulation therapy follow-up, hospitalizations, and emergency visits related to warfarin use) for the pharmacist-managed group was lower than that for the non-pharmacist-managed group ($35,326 vs. $167,412 per 100 patient-years). Additionally, a systematic review encompassing 24 studies indicated that medication reviews conducted by community pharmacists contribute to reduced healthcare costs. In patients with mental illnesses, pharmacist-led medication reviews (including deprescribing) can improve anticholinergic side effects, memory, and quality of life. While pharmacist-led deprescribing does not reduce the consumption of healthcare resources, it does help in saving costs (63).

Recommendation 16: Pharmacists reduce average hospitalization costs through individualized medication therapy and prescription order reviews (I C).

3) Time cost

Average Length of Stay (days) equals the total bed days occupied by discharged patients of a specific disease divided by the total number of discharged patients of the same disease meeting the inclusion criteria during the same period (64).

A systematic review incorporating 18 RCTs and seven economic studies demonstrated that the inclusion of pharmacists in MDTs can reduce hospital stay by an average of 1.74 days (95% CI: −2.76, −0.72), and enhance patient and/or caregiver satisfaction (RR = 1.49, 95% CI: 1.09, 2.03) (65). A meta-analysis indicated that pharmacist interventions in antimicrobial management can reduce the hospital stay of neonates diagnosed or suspected with sepsis requiring antimicrobial treatment (OR = −0.61, 95% CI: −1.86, −1.37, *P* < 0.0001) (66).

Recommendation 17: Pharmacists shorten hospital stays by participating MDTs and intervening in antimicrobial stewardship (I A).

#### 4.2.3 Humanistic value

(1) Patient satisfaction

Patient satisfaction serves as a pivotal metric for evaluating the quality of healthcare services, significantly reflecting the extent to which patients value the care they receive (67). High levels of patient satisfaction are associated with a greater likelihood of continued engagement with beneficial services and adherence to prescribed treatments, thereby fostering a positive feedback loop. This process ultimately enhances outcome indicators and improves overall quality of life (68). Satisfaction assessments may be conducted through various methods, including questionnaires, telephone interviews, WeChat communication, and face-to-face interviews. The evaluation criteria typically include aspects such as treatment effectiveness/problem resolution, service attitude, professional competence, responsiveness, and response time (69). Through patient satisfaction evaluations, we can gain insights into patients' needs, opinions, and suggestions, allowing for timely improvements in pharmaceutical services to enhance service quality and patient experiences. A study assessed patients' perceptions, experiences, and overall satisfaction with pharmacist consultations during oral anticancer drug therapy using a 5-point Likert scale (69). The results indicated that 96.1% of patients were satisfied with the services provided by pharmacists, 93.4% believed that pharmacists offered essential services in outpatient cancer care, and 64.9% of patients felt they had a clearer understanding of the use of oral anticancer drugs and the management of ADRs following pharmacist consultations. This study highlights the value of pharmaceutical counseling services for patients undergoing oral anticancer therapy. Another study investigated patient satisfaction with medication management interventions for heart failure patients using a six-dimensional questionnaire, including treatment efficacy, ease of use, adverse reaction impact, healthcare, effect on daily activities, and overall satisfaction (70). The results indicated a patient satisfaction score of 80.35%.

Recommendation 18: Pharmacists can enhance patient satisfaction through medication counseling, medication education, and MTM (I B).

(2) Healthcare professional satisfaction

The satisfaction of healthcare professionals with pharmacists depends on multiple factors, including the pharmacist's expertise, work efficiency, communication skills, and service attitude. By evaluating healthcare professionals' satisfaction, we can gain insights into the needs, opinions, and suggestions of physicians, nurses, and other medical professionals, allowing for timely improvements in pharmaceutical services and promoting effective collaboration among medical, pharmaceutical, and nursing staff. A tertiary hospital conducted a study to assess caregivers' satisfaction with the pharmaceutical department through a questionnaire survey. Satisfaction levels were categorized into four grades: very satisfied, satisfied, neutral, and dissatisfied, assigned values of 4, 3, 2, and 1, respectively (71). The main evaluation criteria for the pharmaceutical department included the timeliness of drug provision, accuracy of dispensing, availability of consultation services, and involvement in clinical rounds or discussions. The results indicated a high level of satisfaction among clinical healthcare professionals with the pharmaceutical department, with a score of (3.70 ± 0.51). A longitudinal, interventional prospective study investigated healthcare professionals' satisfaction with pharmacists' involvement in medication management for hospitalized older adults with polypharmacy (72). Pharmacists conducted medication reconciliation, optimized medication regimens, and provided medication education. The results indicated a satisfaction rate of 95.9% among healthcare professionals, with 65.3% being very satisfied and 30.6% being satisfied. This outcome supports the value of pharmacist-led MTM in high-risk older populations.

Recommendation 19: Pharmacists enhance healthcare professionals' satisfaction through medication reconciliation, regimen optimization, pharmaceutical rounds, medication education, drug consultations, and case discussions (I C).

(3) Patient trust

Patient trust is crucial as it directly affects medication safety and efficacy. Various scales can quantify patient trust in pharmacists, such as the Trust in the Pharmacist-Patient Relationship Scale (73, 74) and the Multidimensional Trust Scale in Healthcare Systems (75). In a RCT study aimed at reducing emergency and hospitalization rates among home-dwelling diabetes patients through MDT interventions, teams that included pharmacist interventions were more successful in gaining patient trust in the provided healthcare services (76). In an evaluation of veterans' experiences with comprehensive medication management by pharmacists, which included services such as primary care, mental health, pain management, substance use disorder management, and anticoagulation therapy, veterans demonstrated a trust level of 91.9% toward the pharmacists (77).

Recommendation 20: Pharmacists enhance patient trust through chronic disease MTM and pain management (I B).

## 5 Discussion

Clinical pharmacy is a patient-centered discipline focused on rational medication to optimize patient treatment outcomes. According to the American College of Clinical Pharmacy (ACCP), it encompasses the science and practice of rational drug use (78). In terms of clinical value assessment, clinical pharmacists leverage their pharmaceutical expertise to explore patterns of drug use, integrate clinical practice, and achieve optimal disease prevention and treatment. Through services such as pharmaceutical care, medication education, drug consultations, and medication reconciliation, clinical pharmacists significantly enhance patient adherence and achievement of treatment goals, and reduce readmission rates, ADRs, and medication errors, thereby minimizing MRPs and improving therapeutic outcomes. From an economic perspective, while pharmaceutical services may increase direct costs, they ultimately reduce overall treatment expenditures by optimizing treatment regimens and improving therapeutic outcomes, avoiding unnecessary medical costs and preventing potential secondary costs. This results demonstrates favorable cost-effectiveness, cost-utility, and benefit-cost advantages. Regarding humanistic value, pharmacists enhance patient satisfaction and trust through services such as medication counseling, medication education, and medication therapy management. Additionally, pharmacists can improve healthcare professionals' satisfaction through services including medication reconciliation, optimization of medication regimens, pharmaceutical rounds, medication education, drug consultations, and case discussions.

The “Practice Guidelines for the Value Evaluation of Clinical Pharmacy Services (Version 2)” is of significant importance in the field of clinical pharmacy services. It provides a comprehensive set of indicators for evaluating the value of clinical pharmacy services and plays a critical role in promoting the quality and the efficiency of pharmacy services. By offering a scientifically grounded evaluation tool, the guide facilitates the scientific approach to medical decision-making, making the assessment of pharmacy services in healthcare institutions more objective, which in turn, optimizes pharmacy services, improves the safety and efficacy of medication, enhances patient satisfaction and trust, and ultimately elevates the overall patient experience with healthcare services. Additionally, by emphasizing the importance of pharmacy services in resource allocation, the guide aids in the rational planning and utilization of limited medical resources, improving the overall efficiency of healthcare services. Finally, grounded in international advanced pharmacy service concepts and practices, the guide encouraging innovation in existing pharmacy service models to meet the evolving healthcare needs and technological advancements, thereby maintaining the vitality and foresight of pharmacy services.

With the continuous improvement of healthcare policies and the rapid advancement of medical science and technology, the introduction of this guideline comes at a timely moment. We hope that it will contribute to the development of domestic standards for evaluating the effectiveness of clinical pharmacy services and provide a unified framework for the assessment and improvement of pharmacy services. Compared to the first edition, the second edition has been updated with newly included literature, offering the latest clinical evidence and research progress, making the content of the guideline more aligned with current medical practice and patient needs. However, with the ongoing advancement of medical science and technology, and the continuous innovation of clinical pharmacy service models, the content of this guideline may have certain limitations. Advances in medical technology introduce new drugs, treatments, and clinical practices, necessitating regular updates and improvements to the evaluation criteria to accommodate these changes and new requirements. The guideline will evolve in response to new research findings and practical experiences, continuously refining the value assessment system. It will also incorporate feedback and suggestions from peers to perpetually optimize its content and format.

## 6 Joint Committee on Practice Guidelines

### 6.1 Guideline revision steering committee (Listed in alphabetical order by the initials of their surnames)

Jianhong Chen, Army Medical Center of the PLA (People's Liberation Army); Shicai Chen, Beijing Luhe Hospital of Capital Medical University; Weihong Chen, Shanxi Baiqun Hospital (Shanxi Medical Science Academy); Xiao Chen, The First Affiliated Hospital of Sun Yat-Sen University; Deshi Dong, The First Affiliated Hospital of Dalian Medical University; Weihong Ge, Nanjing Drum Tower Hospital, The Affiliated Hospital of Nanjing University Medical School; Ruichen Guo, Qilu Hospital of Shandong University; Yuechuan Jia, General Hospital of Ningxia Medical University; Guohui Li, Cancer Institute and Hospital of Chinese Academy of Medical Sciences; Huande Li, The Second Xiangya Hospital of Central South University; Pengmei Li, China-Japan Friendship Hospital; Gaolin Liu, Shanghai General Hospital; Lihong Liu, China-Japan Friendship Hospital; Shiting Liu, Nanfang Hospital of Southern Medical University; Xiaoyang Lu, The First Affiliated Hospital of College of Medicine of Zhejiang University; Manling Ma, The Sixth Affiliated Hospital of Harbin Medical University; Feng Qiu, The First Affiliated Hospital of Chongqing Medical University; Aizong Shen, China Science and Technology University Affiliated First Hospital; Yanqing Song, First Hospital of Jilin University; Jiawei Wang, Beijing Tongren Hospital of Capital Medical University; Dongfang Wu, Zhongnan Hospital of Wuhan University; Xinan Wu, The First Hospital of Lanzhou University; Peiyuan Xia, First Affiliated Hospital of Third Military Medical University; Juan Xie, Guizhou Provincial People's Hospital; Bikui Zhang, The Second Xiangya Hospital of Central South University; Bo Zhang, Peking Union Medical College Hospital; Jian Zhang, Xinhua Hospital Affiliated to Shanghai Jiao Tong University School of Medicine; Lan Zhang, Xuanwu Hospital of Capital Medical University; Lingli Zhang, West China Second Hospital of Sichuan University, West China Women's and Children's Hospital; Yu Zhang, Department of Pharmacy, Union Hospital, Tongji Medical College, Huazhong University of Science and Technology; Limei Zhao, Shengjing Hospital of China Medical University; Jie Zhao, The First Affiliated Hospital of Zhengzhou University; Rongsheng Zhao, Peking University Third Hospital.

### 6.2 Expert group for guideline revision (Listed in alphabetical order by the initials of their surnames)

Basang Lhamo, People's Hospital of the Tibet Autonomous Region; Yalin Dong, The First Affiliated Hospital of Xi'an Jiaotong University; Daihong Guo, Chinese PLA General Hospital; Jinyu Guo, Jining No.1 People's Hospital; Jinhan Hou, West China Hospital, Sichuan University; Ruigang Hou, The Second Hospital of Shanxi Medical University; Baorong Hu, The First Affiliated Hospital of Harbin Medical University; Ming Hu, West China School of Pharmacy, Sichuan University; Yilan Huang, The Affiliated Hospital of Southwest Medical University; Yuechuan Jia, General Hospital of Ningxia Medical University; Lingyan Jian, Shengjing Hospital of China Medical University; Ling Jiang, The First Affiliated Hospital of University of Science and Technology of China; Guohui Li, Cancer Hospital Chinese Academy of Medical Sciences; Shuhong Liang, The First Affiliated Hospital of Zhengzhou University; Dong Liu, Tongji Hospital, Huazhong University of Science and Technology; Jingfeng Liu, Fujian Cancer Hospital; Dan Mei, Peking Union Medical College Hospital; Liyan Miu, The First Affiliated Hospital of Soochow University; Xiaoyong Qi, Hebei General Hospital; Yan Qian, The Second Affiliated Hospital of Chongqing Medical University; Feng Qiu, The First Affiliated Hospital of Chongqing Medical University; Chen Shi, Department of Pharmacy, Union Hospital, Tongji Medical College, Huazhong University of Science and Technology; Zhouliang Sun, The Third Hospital of Xiamen (The First Affiliated Hospital of Xiamen University Tongan District); Jianhua Wang, The First Affiliated Hospital of Xinjiang Medical University; Aidong Wen, Xijing Hospital, Air Force Medical University; Dongfang Wu, Zhongnan Hospital of Wuhan University; Fengbo Wu, West China Hospital, Sichuan University; Xuan Xiong, Sichuan Academy of Medical Sciences and Sichuan Provincial People's Hospital; Feifei Xu, Sichuan Academy of Medical Sciences and Sichuan Provincial People's Hospital; Jiadan Yang, The First Affiliated Hospital of Chongqing Medical University; Yong Yang, Sichuan Academy of Medical Sciences and Sichuan Provincial People's Hospital; Qian Yu, China-Japan Union Hospital of Jilin University; Hongtao Xiao, Sichuan Cancer Hospital; Bikui Zhang, The Second Xiangya Hospital of Central South University; Kanghuai Zhang, The Second Affiliated Hospital of Xi'an Jiaotong University; Wei Zhang, Henan Provincial People's Hospital; Xingguo Zhang, Beilun Branch of the First Affiliated Hospital of Zhejiang University School of Medicine; Zhiren Zhang, The First Affiliated Hospital of Harbin Medical University; Zhigang Zhao, Beijing Tiantan Hospital, Capital Medical University; Changyu Zhu, Sichuan Academy of Medical Sciences and Sichuan Provincial People's Hospital; Xiaocong Zuo, The Third Xiangya Hospital of Central South University.

## Data Availability

The original contributions presented in the study are included in the article/supplementary material, further inquiries can be directed to the corresponding author.

## References

[1] The Central People's Government of the People's Republic of China. Opinions of the Central Committee of the Communist Party of China and the State Council on Deepening the Reform of the Medical and Health System [M/OL] (2009). Available at: https://www.gov.cn/jrzg/2009-04/06/content_1278721.htm.

[2] Office Office of the National Health and Family Planning Commission State State Administration of Traditional Chinese Medicine. Notice on Strengthening Pharmaceutical Management and Transforming Pharmaceutical Service Models, Guo Wei Ban Yi Fa [2017] No. 26 [M/OL] (2017). Available at: http://www.nhc.gov.cn/yzygj/s7659/201707/b44339ebef924f038003e1b7dca492f2.shtml

[3] National Health Commission State State Administration of Traditional Chinese Medicine. Opinions on Accelerating the High Quality Development of Pharmaceutical Services [M/OL] (2018). Available at: https://www.gov.cn/zhengce/zhengceku/2018-12/31/content_5436829.htm

[4] National Health Commission Ministry Ministry of Finance National Medical Insurance Administration Ministry Ministry of Education Ministry Ministry of Human Resources and Social Security . Notice on Issuing Opinions on Strengthening Drug Administration in Medical Institutions and Promoting Rational Drug Use [M/OL] (2020). Available at: http://www.nhc.gov.cn/yzygj/s7659/202002/ea3b96d1ac094c47a1fc39cf00f3960e.shtml

[5] Li X Wu Y. Guidelines for the Work of Clinical Pharmacists, 2nd Edn. Beijing: People's Medical Publishing House (2012).

[6] Chen H Ma Z Liu B Zhong X Zhang J. Bibliometric analysis of entry points for clinical pharmacy work based on PubMed database. Eval Anal Drug Use Hosp China. (2024) 24:5069. 10.14009/j.issn.1672-2124.2024.04.028

[7] Martínez-Mardones F Fernandez-Llimos F Benrimoj SI Ahumada-Canale A Plaza-Plaza JC Tonin FS . Systematic review and meta-analysis of medication reviews conducted by pharmacists on cardiovascular diseases risk factors in ambulatory care. J Am Heart Assoc. (2019) 8:e013627. 10.1161/JAHA.119.013627 31711390 PMC6915276

[8] Nakanishi M Mizuno T Mizokami F Koseki T Takahashi K Tsuboi N . Impact of pharmacist intervention for blood pressure control in patients with chronic kidney disease: a meta-analysis of randomized clinical trials. J Clin Pharm Ther. (2021) 46:114–20. 10.1111/jcpt.13262 32949161

[9] Ramsubeik K Ramrattan LA Kaeley GS Singh JA. Effectiveness of healthcare educational and behavioral interventions to improve gout outcomes: a systematic review and meta-analysis. Ther Adv Musculoskelet Dis. (2018) 10:235–52. 10.1177/1759720X18807117 30515250 PMC6262501

[10] Delage C Lelong H Brion F Blacher J. Effect of a pharmacist-led educational intervention on clinical outcomes: a randomised controlled study in patients with hypertension, type 2 diabetes and hypercholesterolaemia. Eur J Hosp Pharm. (2021) 28:e197–202. 10.1136/ejhpharm-2021-002787 34183458 PMC8640406

[11] Zhang Q Su H Li B Bai X Yan S Li X. Physician-pharmacist collaborative management in patients after percutaneous coronary intervention: a retrospective propensity score matching cohort study. Int J Clin Pharm. (2022) 44:90–9. 10.1007/s11096-021-01316-0 34643858

[12] Kiel PJ Mccord AD. Pharmacist impact on clinical outcomes in a diabetes disease management program via collaborative practice. Ann Pharmacother. (2005) 39:1828–32. 10.1345/aph.1G356 16219894

[13] Howard-Thompson A Farland MZ Byrd DC Airee A Thomas J Campbell J . Pharmacist-physician collaboration for diabetes care: cardiovascular outcomes. Ann Pharmacother. (2013) 47:1471–7. 10.1177/1060028013504738 24285763

[14] Kelly WN Ho MJ Bullers K Klocksieben F Kumar Kumar A Association of pharmacist counseling with adherence 30-day readmission and and mortality: a systematic review and meta-analysis of randomized trials. J Am Pharm Assoc. (2021) 61:340–50.e5. 10.1016/j.japh.2021.01.028 33678564

[15] Bonetti AF Reis WC Mendes AM Rotta I Tonin FS Fernandez-Llimos F . Impact of pharmacist-led discharge counseling on hospital readmission and emergency department visits: a systematic review and meta-analysis. J Hosp Med. (2020) 15:52–9. 10.12788/jhm.3182 30897055

[16] Ruiz-Ramos J Hernández MH Juanes-Borrego AM Milà R Mangues-Bafalluy MA Mestres C. The impact of pharmaceutical care in multidisciplinary teams on health outcomes: systematic review and meta-analysis. J Am Med Dir Assoc. (2021) 22:2518–26. 10.1016/j.jamda.2021.05.038 34228962

[17] Harris M Moore V Barnes M Persha H Reed J Zillich A. Effect of pharmacy-led interventions during care transitions on patient hospital readmission: a systematic review. J Am Pharm Assoc. (2022) 62:1477–98.e8. 10.1016/j.japh.2022.05.017 35718715

[18] Mckay C Park C Chang J Brackbill M Choi JY Lee JH . Systematic review and meta-analysis of pharmacist-led transitions of care services on the 30-day all-cause readmission rate of patients with congestive heart failure. Clin Drug Investig. (2019) 39:703–12. 10.1007/s40261-019-00797-2 31102109

[19] Ali S Salahudeen MS Bereznicki LRE Curtain CM. Pharmacist-led interventions to reduce adverse drug events in older people living in residential aged care facilities: a systematic review. Br J Clin Pharmacol. (2021) 87:3672–89. 10.1111/bcp.14824 33880786

[20] Lee H Ryu K Sohn Y Kim J Suh GY Kim E. Impact on patient outcomes of pharmacist participation in multidisciplinary critical care teams: a systematic review and meta-analysis. Crit Care Med. (2019) 47:1243–50. 10.1097/CCM.0000000000003830 31135496

[21] Ciapponi A Fernandez Nievas SE Seijo M Rodríguez MB Vietto V García-Perdomo HA . Reducing medication errors for adults in hospital settings. Cochrane Database Syst Rev. (2021) 11:CD009985. 10.1002/14651858.CD009985.pub2 34822165 PMC8614640

[22] De Oliveira GS Jr Castro-Alves LJ Kendall MC McCarthy R. Effectiveness of pharmacist intervention to reduce medication errors and health-care resources utilization after transitions of care: a meta-analysis of randomized controlled trials. J Patient Saf. (2021) 17:375–80. 10.1097/PTS.0000000000000283 28671909

[23] Naseralallah LM Hussain TA Jaam M Pawluk SA. Impact of pharmacist interventions on medication errors in hospitalized pediatric patients: a systematic review and meta-analysis. Int J Clin Pharm. (2020) 42:979–94. 10.1007/s11096-020-01034-z 32328958

[24] Yates L Valente M Wadsworth C. Evaluation of pharmacist medication review service in an outpatient heart failure clinic. J Pharm Pract. (2020) 33:820–6. 10.1177/0897190019842696 31057060

[25] Moukafih B Abahssain H Mrabti H Errihani H Rahali Y Taoufik J . Impact of clinical pharmacy services in a hematology/oncology ward in Morocco. J Oncol Pharm Pract. (2021) 27:305–11. 10.1177/1078155220919169 32326873

[26] Zhang Y Yang H Kong J Liu L Ran L Zhang X . Impact of interventions targeting the inappropriate use of proton-pump inhibitors by clinical pharmacists in a hepatobiliary surgery department. J Clin Pharm Ther. (2021) 46:149–57. 10.1111/jcpt.13273 33015848

[27] Zeng Y Zheng Z. American Pharmacists Association Medication Therapy Management Services. Beijing: China Medical Science and Technology Press (2018), p. 11–4.

[28] Halvorsen KH Stadelokken T Garcia BH. A stepwise pharmacist-led medication review service in interdisciplinary teams in rural nursing homes. Pharmacy. (2019) 7:148. 10.3390/pharmacy7040148 31694298 PMC6958343

[29] Liu J Wang C Chen X Luo J Xie J Li S . Evaluation of pharmacist interventions as part of a multidisciplinary cancer pain management team in a Chinese academic medical center. J Am Pharm Assoc. (2020) 60:76–80. 10.1016/j.japh.2019.09.005 31669418

[30] Morisky DE Green LW Levine DM. Concurrent and predictive validity of a self-reported measure of medication adherence. Med Care. (1986) 24:67–74. 10.1097/00005650-198601000-00007 3945130

[31] Morisky DE Ang A Krousel-Wood M Ward HJ. Predictive validity of a medication adherence measure in an outpatient setting. J Clin Hypertens. (2008) 10:348–54. 10.1111/j.1751-7176.2008.07572.x 18453793 PMC2562622

[32] Kim MT Hill MN Bone LR Levine DM. Development and testing of the hill-bone compliance to high blood pressure therapy scale. Prog Cardiovasc Nurs. (2000) 15:90–6. 10.1111/j.1751-7117.2000.tb00211.x 10951950

[33] Thompson K Kulkarni J Sergejew AA. Reliability and validity of a new Medication Adherence Rating Scale (MARS) for the psychoses. Schizophr Res. (2000) 42:241–7. 10.1016/S0920-9964(99)00130-9 10785582

[34] Jia X Zhou S Luo D Zhao X Zhou Y Cui YM. Effect of pharmacist-led interventions on medication adherence and inhalation technique in adult patients with asthma or COPD: a systematic review and meta-analysis. J Clin Pharm Ther. (2020) 45:904–17. 10.1111/jcpt.13126 32107837

[35] Presley B Groot W Pavlova M. Pharmacy-led interventions to improve medication adherence among adults with diabetes: a systematic review and meta-analysis. Res Social Adm Pharm. (2019) 15:1057–67. 10.1016/j.sapharm.2018.09.021 30685443

[36] Shawahna R Thawabi F Salah R Ramadan S. Pharmaceutical care services for patients with diabetes: a systematic scoping review. Am J Manag Care. (2022) 28:e339–46. 10.37765/ajmc.2022.89227 36121366

[37] Liu X Yan J Han L Han Y Shen Q Du S . Specification for emergency remote pharmacy services for the prevention and control of COVID-19 pneumonia. Chin Med. (2020) 15:987–91. Available at: http://link–cnki–net–https.cnki.scrm.scsycy.vip:2222/urlid/11.5451.R.20200622.1111.008

[38] Cole J Wilkins N Moss M Fu D Carson P Xiong L. Impact of Pharmacist Involvement on Telehealth Transitional Care Management (TCM) for High Medication Risk Patients. Pharmacy. (2019) 7:158. 10.3390/pharmacy7040158 31775263 PMC6958334

[39] Liu G. Guidelines for Pharmacoeconomic Evaluation in China. Beijing: China Market Press (2020).

[40] Polgreen LA Han J Carter BL Ardery GP Coffey CS Chrischilles EA . Cost-effectiveness of a physician-pharmacist collaboration intervention to improve blood pressure control. Hypertension. (2015) 66:1145–51. 10.1161/HYPERTENSIONAHA.115.06023 26527048 PMC4644092

[41] Simpson SH Lier DA Majumdar SR Tsuyuki RT Lewanczuk RZ Spooner R . Cost-effectiveness analysis of adding pharmacists to primary care teams to reduce cardiovascular risk in patients with type 2 diabetes: results from a randomized controlled trial. Diabet Med. (2015) 32:899–906. 10.1111/dme.12692 25594919

[42] Shi R Shen Z Shi Y Wei Y. Efficacy and pharmacoeconomic evaluation of clinical pharmacists involved in the treatment of community-acquired pneumonia. J Guangdong Pharm Univ. (2021) 37:60–4. 10.16809/j.cnki.2096-3653.2021040905

[43] D'hulster E Quintens C Bisschops R Willems R Peetermans WE Verbakel JY . Cost-effectiveness of check of medication appropriateness: methodological approach. Int J Clin Pharm. (2022) 44:399–408. 10.1007/s11096-021-01356-6 35013878

[44] Ahumada-Canale A Quirland C Martinez-Mardones FJ Plaza-Plaza JC Benrimoj S Garcia-Cardenas V. Economic evaluations of pharmacist-led medication review in outpatients with hypertension, type 2 diabetes mellitus, and dyslipidaemia: a systematic review. Eur J Health Econ. (2019) 20:1103–16. 10.1007/s10198-019-01080-z 31218580

[45] Sanyal C Turner JP Martin P Tannenbaum C. Cost-effectiveness of pharmacist-led deprescribing of NSAIDs in community-dwelling older adults. J Am Geriatr Soc. (2020) 68:1090–7. 10.1111/jgs.16388 32105355

[46] Kulchaitanaroaj P Brooks JM Chaiyakunapruk N Goedken AM Chrischilles EA Carter BL. Cost-utility analysis of physician-pharmacist collaborative intervention for treating hypertension compared with usual care. J Hypertens. (2017) 35:178–87. 10.1097/HJH.0000000000001126 27684354

[47] Tam-Tham H Clement F Hemmelgarn BR Manns BJ Klarenbach SW Tonelli M . A cost analysis and cost-utility analysis of a community pharmacist-led intervention on reducing cardiovascular risk: the alberta vascular risk reduction community pharmacy project (R(x)EACH). Value Health. (2019) 22:1128–36. 10.1016/j.jval.2019.05.012 31563255

[48] Tanaka K Tachi T Hori A Osawa T Nagaya K Makino T . Cost utility analysis of pharmacist counseling care for breast cancer chemotherapy outpatients. Pharmazie. (2019) 74:439–42. 10.1691/ph.2019.9327 31288902

[49] Munoz-Pichuante D Villa-Zapata L. Benefit of incorporating clinical pharmacists in an adult intensive care unit: a cost-saving study. J Clin Pharm Ther. (2020) 45:1127–33. 10.1111/jcpt.13195 32497354

[50] Al-Qudah R A Al-Badriyeh D Al-Ali FM Altawalbeh SM Basheti IA. Cost-benefit analysis of clinical pharmacist intervention in preventing adverse drug events in the general chronic diseases outpatients. J Eval Clin Pract. (2020) 26:115–24. 10.1111/jep.13209 31234234

[51] Chen M Zhang L Zhang C Huang L Hu Z. Cost benefit analysis of pediatric clinical pharmacist pharmaceutical services. China Pharmacy. (2018) 29:483−6.

[52] Freeman CR Scott IA Hemming K Connelly LB Kirkpatrick CM Coombes I . Reducing Medical Admissions and Presentations Into Hospital through Optimising Medicines (REMAIN HOME): a stepped wedge, cluster randomised controlled trial. Med J Aust. (2021) 214:212–7. 10.5694/mja2.50942 33580553

[53] Taber DJ Fleming JN Su Z Mauldin P McGillicuddy JW Posadas A . Significant hospitalization cost savings to the payer with a pharmacist-led mobile health intervention to improve medication safety in kidney transplant recipients. Am J Transplant. (2021) 21:3428–35. 10.1111/ajt.16737 34197699

[54] Wilkes S Zaal RJ Abdulla A Hunfeld NGM. A cost-benefit analysis of hospital-wide medication reviews: a period prevalence study. Int J Clin Pharm. (2022) 44:138–45. 10.1007/s11096-021-01323-1 34498214 PMC8866269

[55] Kong L Wang H Peng M Qiu F Yang J Shan X. Discussion on the economic value of pharmaceutical services in medical institutions based on cost-benefit analysis. Chin Pharmacy. (2022) 33:1769−75.

[56] Ma Z Zhao Z Sun S Li Y An Z Yan Y . Impact of ‘chief-pharmacist system' on drug expenditures and rational drug use. Int J Clin Pharm. (2020) 42:167–73. 10.1007/s11096-019-00954-9 31919733

[57] General Office of the National Health Commission. Notice from the General Office of the National Health Commission on Issuing the Performance Appraisal Operation Manual for National Third level Public Hospitals (2023 Edition). Beijing (2023).

[58] Zhang K Lin Y Wang F Chen W. A brief discussion on pharmaceutical intervention for unreasonable drug use in hospitals by the pharmacy department. Anhui Pharm. (2019) 23:390–5. Available at: http://link–cnki–net–https.cnki.scrm.scsycy.vip:2222/urlid/34.1229.R.20190131.1028.100

[59] Gong Y Chen Q Zhang Y. The role of the clinical pharmacist on the health outcomes of acute exacerbations of chronic obstructive pulmonary disease (AECOPD). Int J Chron Obstruct Pulmon Dis. (2022) 17:1863–70. 10.2147/COPD.S370532 35996393 PMC9391938

[60] Chan FW Wong RS Lau WH Chan TY Cheng G You JH. Management of Chinese patients on warfarin therapy in two models of anticoagulation service - a prospective randomized trial. Br J Clin Pharmacol. (2006) 62:601–9. 10.1111/j.1365-2125.2006.02693.x 17061966 PMC1885165

[61] Hall D Buchanan J Helms B Eberts M Mark S Manolis C . Health care expenditures and therapeutic outcomes of a pharmacist-managed anticoagulation service versus usual medical care. Pharmacotherapy. (2011) 31:686–94. 10.1592/phco.31.7.686 21923456

[62] Hou K Yang H Ye Z Wang Y Liu L Cui X. Effectiveness of pharmacist-led anticoagulation management on clinical outcomes: a systematic review and meta-analysis. J Pharm Pharm Sci. (2017) 20:378–96. 10.18433/J3SQ0B 29145935

[63] Buzancic I Kummer I Drzaic M Ortner HadŽiabdić M. Community-based pharmacists' role in deprescribing: a systematic review. Br J Clin Pharmacol. (2022) 88:452–63. 10.1111/bcp.14947 34155673

[64] NHC Office. Operational Manual for Performance Assessment of Tertiary Public Hospitals (2024 Edition). (2024). Available at: http://www.nhc.gov.cn/yzygj/s3594q/202403/94a97921a9b043e8b8e3315aed9f1627.shtml

[65] Dawoud DM Smyth M Ashe J Strong T Wonderling D Hill J . Effectiveness and cost effectiveness of pharmacist input at the ward level: a systematic review and meta-analysis. Res Social Adm Pharm. (2019) 15:1212–22. 10.1016/j.sapharm.2018.10.006 30389320

[66] Chen C Tan S Deng M. Meta analysis of the impact of clinical pharmacist intervention in antimicrobial drug management on neonatal blood borne infections. Chin Med Innov. (2021) 18:164–70.

[67] Garman AN Garcia J Hargreaves M. Patient satisfaction as a predictor of return-to-provider behavior: analysis and assessment of financial implications. Qual Manag Health Care. (2004) 13:75–80. 10.1097/00019514-200401000-00007 14976909

[68] Oluwole EO Osibogun O Adegoke O Adejimi AA Adewole AM Osibogun A . Medication adherence and patient satisfaction among hypertensive patients attending outpatient clinic in Lagos University Teaching Hospital, Nigeria. Niger Postgrad Med J. (2019) 26:129–37. 10.4103/npmj.npmj_48_19 31187754

[69] Dennis M Haines A Johnson M Soggee J Tong S Parsons R . Cross-sectional census survey of patients with cancer who received a pharmacist consultation in a pharmacist led anti-cancer clinic. J Cancer Educ. (2022) 37:1553–61. 10.1007/s13187-022-02196-2 35867307 PMC9305046

[70] Wondesen A Berha AB Woldu M Mekonnen D Engidawork E. Impact of medication therapy management interventions on drug therapy problems, medication adherence and treatment satisfaction among ambulatory heart failure patients at Tikur Anbessa Specialised Hospital, Addis Ababa, Ethiopia: a one-group pre-post quasi-experimental study. BMJ Open. (2022) 12:e054913. 10.1136/bmjopen-2021-054913 35414550 PMC9006832

[71] Kong X Wang J Geng L Gao T Cheng C. Analysis of satisfaction evaluation of clinical medical staff in a tertiary hospital towards the medical technology department. Chin J Soc Med. (2020) 37:677–81.

[72] Ruiz-Millo O Climente-Marti M Navarro-Sanz JR. Patient and health professional satisfaction with an interdisciplinary patient safety program. Int J Clin Pharm. (2018) 40:635–41. 10.1007/s11096-018-0627-7 29594676

[73] Lin J Liu X An C Zhu M. Development and reliability and validity testing of a drug patient relationship trust measurement scale. Chin Hosp Pharm J. (2019) 39:1203–6. 10.13286/j.cnki.chinhosppharmacyj.2019.11.20

[74] Ding J Zhou J Ren W. Construction and application research of drug safety trust evaluation index system. Med Guide. (2020) 39:875–9.

[75] Egede L E Ellis C. Development and testing of the multidimensional trust in health care systems scale. J Gen Intern Med. (2008) 23:808–15. 10.1007/s11606-008-0613-1 18415653 PMC2517872

[76] Casten R Rovner B Chang AM Hollander JE Kelley M Leiby B . A randomized clinical trial of a collaborative home-based diabetes intervention to reduce emergency department visits and hospitalizations in black individuals with diabetes. Contemp Clin Trials. (2020) 95:106069. 10.1016/j.cct.2020.106069 32561466

[77] McFarland MS Tran M Ourth HL Morreale AP. Evaluation of patient experience with veterans affairs clinical pharmacist practitioners providing comprehensive medication management. J Pharm Pract. (2023) 36:1356–61. 10.1177/08971900221117892 35924640

[78] American College of Clinical Pharmacy. The definition of clinical pharmacy. Pharmacotherapy. (2008) 28:816–7. 10.1592/phco.28.6.816 18503408

